# Effect of magnetic stimulation of Shenmen point on cognitive function of chronic insomnia: A randomized controlled clinical trial

**DOI:** 10.1097/MD.0000000000023807

**Published:** 2020-12-18

**Authors:** Jie Yuan, Yimeng Chen, Penglong Yu, Fan Luo, Yongxiang Gao, Jie Chen, Pei Wang, Yuan Wang, Yuan Zhao, Yaling Lei

**Affiliations:** a School of Basic Medical Sciences, Chengdu University of Traditional Chinese Medicine, Chengdu; b Department of Encephalopathy, Shaanxi Provincial Hospital of Traditional Chinese Medicine, Xi’an; c Adult rehabilitation Department, the First People's Hospital of Yinchuan, Yinchuan; d Hospital of Chengdu University of Traditional Chinese Medicine; e International Education College, Chengdu University of Traditional Chinese Medicine, Jiuniu District, Chengdu, Sichuan, China.

**Keywords:** chronic insomnia, clinical trial, HT7, repetitive magnetic stimulation, repetitive transcranial magnetic stimulation, research protocol

## Abstract

**Background:**

Chronic insomnia (CI) can lead to cognitive dysfunction and bring great pain to patients’ life. There is no effective intervention for cognitive dysfunction caused by CI. Shenmen (HT7) is the first choice for insomnia treatment. However, the effect and mechanism of this acupoint on cognitive function after insomnia is not clear. Therefore, the purpose of this study is to explore whether magnetic stimulation of HT7 can improve cognitive impairment of CI by regulating prefrontal lobe and its mechanism.

**Methods/Design:**

This is a randomized controlled clinical trial. Seventy-two subjects aged 18 to 65 years old with primary insomnia and more than 3 months were randomly divided into 2 groups according to the ratio of 1:1, and 36 healthy controls were included. The control group was given sleep hygiene and cognitive therapy in behavioral cognitive therapy technology, while the experimental group was given the behavioral cognitive therapy technology intervention and magnetic stimulation of HT7 acupoint for 30 times (2 times / d, 5 times / wk for 20 days), while the healthy control group had no intervention measures. Before treatment and 20 days after treatment, we evaluated the working memory (1-back test), episodic memory (Complex Figure Test), and problem-solving ability (Hanoi tower test) processed by prefrontal lobe to explore the effect of magnetic stimulation on cognitive function of CI and its possible mechanism. At the same time, insomnia severity index was used to evaluate sleep state, Becker depression scale was used to evaluate depression, and Beck anxiety scale was used to evaluate anxiety. Chi-squared test or rank sum test was used to collect the data of patients. If *P* value is less than or equal to .05, the difference will be considered statistically significant.

**Conclusion:**

This study explored the effect and mechanism of magnetic stimulation of Shenmen (HT7) on cognitive function of CI, and confirmed that magnetic stimulation of HT7 can be used as an alternative therapy to improve cognitive impairment of CI.

**Trial Registration number:**

ChiCTR2000034280

## 1 Introduction

Chronic insomnia (CI) is a common disease in adults.^[1]^ Long term sleep disorders not only affect the quality of life of patients, but also cause cognitive impairment. Sleep deficiency can cause damage in many cognitive fields such as attention, memory, executive power, emotional control, language fluency, mental motor speed, etc.^[2]^ The worse sleep quality, the more and more areas and degree of cognitive impairment.^[3]^

At present, the cognitive impairment of CI lacks effective intervention measures, and behavioral cognitive therapy (CBT-I) lacks sufficient clinical evidence to improve the cognitive function of CI.^[4]^ Oral hypnotics may aggravate cognitive impairment.^[5]^ The possible mechanism of cognitive impairment in insomnia is increased cortical excitability^[2]^ and decreased slow wave sleep.^[6]^ Repetitive transcranial magnetic stimulation (rTMS) is a noninvasive neuromodulation technique, which is widely used in clinical practice by reducing the cortical excitability of CI patients.^[7]^ However, the use of rTMS in patients with intracranial metal implants, stents, glaucoma, and hypertension is limited.

Acupuncture and moxibustion is one of the common therapies for insomnia in traditional Chinese medicine (TCM). Shenmen (HT7) is the original point of hand Shaoyin heart meridian, which has the effect of calming and calming the mind. It is the preferred acupoint in TCM treatment of insomnia.^[8]^ The clinical effective rate of Shenmen single point in treating primary insomnia is 76.67%.^[9]^ However, it is difficult to copy the manipulation of acupuncture and moxibustion treatment, and some patients can not bear the pain of acupuncture.

Repetitive magnetic stimulation (rMS) acupoint has the similar effect as acupuncture and moxibustion, and has the advantages of quantifiable, non-invasive, and pain free.^[10]^ Compared with rTMS, rMS has fewer contraindications, so the use of rMS to stimulate acupoints to improve CI symptoms has attracted the attention of clinical workers. In clinical evidence, low frequency (1 Hz) rMS of HT7 point can make brain activity orderly and reduce brain excitability to health.^[11]^ Moreover, low frequency (1 Hz) stimulation of HT7, PC6, and SP6 by rMS can enhance the interaction between brain regions, improve the transmission speed and efficiency between brain networks, and improve the cognitive function of brain.^[12]^ However, it is not clear whether magnetic stimulation of HT7 can improve cognitive impairment associated with CI. Therefore, we designed this experiment to explore the short-term efficacy and possible mechanism of rMs stimulating HT7 on cognitive impairment of CI.

## 2 Methods/design

### 2.1 Study design

This is a randomized, parallel controlled clinical trial. A total of 108 volunteers were recruited. Seventy-two patients with CI were randomly divided into 2 groups, the ratio was 1:1. Both groups were given basic treatment (sleep hygiene + cognitive therapy in CBT-I Technology), and the experimental group was added magnetic stimulation of HT7 points on the basis of basic treatment. There were 36 healthy people in the control group. There was no intervention in the healthy control group.

The whole study period was 20 days. The clinical efficacy was evaluated by clinical efficacy scale and related software on the day of enrollment and the 20th day of treatment.

The clinical trial results will be reported according to the Standards for Reporting

Interventions in Clinical Trials of Acupuncture statement.^[13]^

### 2.2 Ethics

This study has passed the ethics committee Shaanxi Provincial Hospital of traditional Chinese medicine ((2020) Lun Shen No. (17)), and registered on June 30, 2020 at http://www.chictr.org.cn/index.aspx, ID is ChiCTR2000034280. Informed consent has included all the subjects involved. Any modification of the study protocol or informed consent, which may affect the rights and interests of the participants or the implementation of the study, should be reported to the Ethics Committee for approval again. If there are any serious adverse events in the trial, the ethics committee should review it in time and put forward written suggestions for modification, including sufficient power to suspend the trial.

### 2.3 Study population

#### 2.3.1 Inclusion criteria of control group and experimental group

1. Patients aged 18 to 45 years, including 18 and 45, with no gender limit;
2. Patients who meet the DSM-5 diagnostic criteria for CI;
3. Patients with insomnia severity index > 7;
4. Chinese handedness scale > = 7 patients;
5. Patients who sign informed consent.

#### 2.3.2 Inclusion criteria of healthy control group

1. Age: 18 to 45 years old;
2. BMI: 18.5 to 23.9 kg/m^2^;
3. Education: secondary school or above (including secondary school);
4. Chinese handedness scale >= 7 patients;
5. mini mental state examination>27 minute;
6. patient health questionnaire-9<5 minute;
7. generalized anxiety disorder-7<5 minute;
8. insomnia severity index < 7 minute.

#### 2.3.3 Exclusion criteria of control group and experimental group

1. Patients with insomnia accompanied by other mental diseases (depression, anxiety, fine score, double equal), physical diseases (pain, stroke, coronary heart disease, tumor, etc);
2. Patients with severe depression and anxiety (patient health questionnaire-9 depression screening >= 20; generalized anxiety disorder-7 anxiety screening >= 19);
3. Patients with insomnia caused by shift work, time difference, and other reasons;
4. Patients with high risk of sleep apnea (the patients with more than 3 items of stop Bang questionnaire were judged as high risk);
5. Patients with severe or unstable chronic diseases that are not well controlled;
6. Patients with central nervous system diseases, such as epilepsy, brain tumor, hemangioma, encephalitis, acute brain injury, etc;
7. Patients with metal foreign bodies within 30 cm of the treatment site;
8. Patients who received transcranial magnetic stimulation (TMS), transcranial direct current stimulation, acupuncture and benzodiazepines regularly within 2 weeks before enrollment;
9. Patients who depend on or abuse alcohol, drugs, and other substances.

#### 2.3.4 Exclusion criteria of healthy control group

1. History of mental illness;
2. Family history of mental illness;
3. History of major trauma;
4. Nervous system diseases (such as: cerebral thrombosis, cerebral infarction, epilepsy, brain tumor, peripheral neuropathy, etc);
5. History of head trauma (e.g., coma, vomiting, amnesia, convulsions, or emergency treatment);
6. Have bad habits such as smoking and drinking;
7. Serious diseases of important organs (such as heart, lung, liver, kidney, etc).

#### 2.3.5 Withdrawal criteria

1. Violation of inclusion criteria and clinical trial protocol or exclusion criteria;
2. Less than 30 sessions of RMS treatment were performed within 20 days;
3. Serious adverse events occurred;
4. Lost follow-up;
5. Withdrawal of consent by subject or legal representative;
6. Increase the treatment that may affect the study without the permission of the investigator.

### 2.4 Study settings and recruitments

This study will be carried out in Shaanxi Provincial Hospital of traditional Chinese medicine.CI subjects were mainly recruited from the wards and outpatient departments of Shaanxi Provincial Hospital of traditional Chinese medicine. At the same time, posters, leaflets, and routine free examinations will also help the recruitment of volunteers. In addition, the recruitment announcement on the official website of the hospital, official account of WeChat, micro-blog and other Internet media will help the volunteers to successfully recruit.

### 2.5 Study group

Seventy-two CI subjects who met the inclusion criteria and signed the informed consent form were randomly divided into the experimental group and the control group according to the ratio of 1:1; 36 healthy people were recruited according to the inclusion criteria of the healthy control group.

### 2.6 Study time

This clinical study will be conducted from August 1, 2020 to August 1, 2022.

### 2.7 Interventions

The intervention measures designed in this study are based on TCM theory and professional practice knowledge. Relevant doctors have obtained the license issued by the national health and Family Planning Commission of China, and have received more than 3 years of clinical skills training in acupuncture and TMS. Patients in the experimental group and the control group were given basic treatment (sleep hygiene and cognitive therapy in CBT-I Technology); in addition, patients in the experimental group were given rMS treatment at HT7.

#### 2.7.1 Experimental group

**Figure 1:**
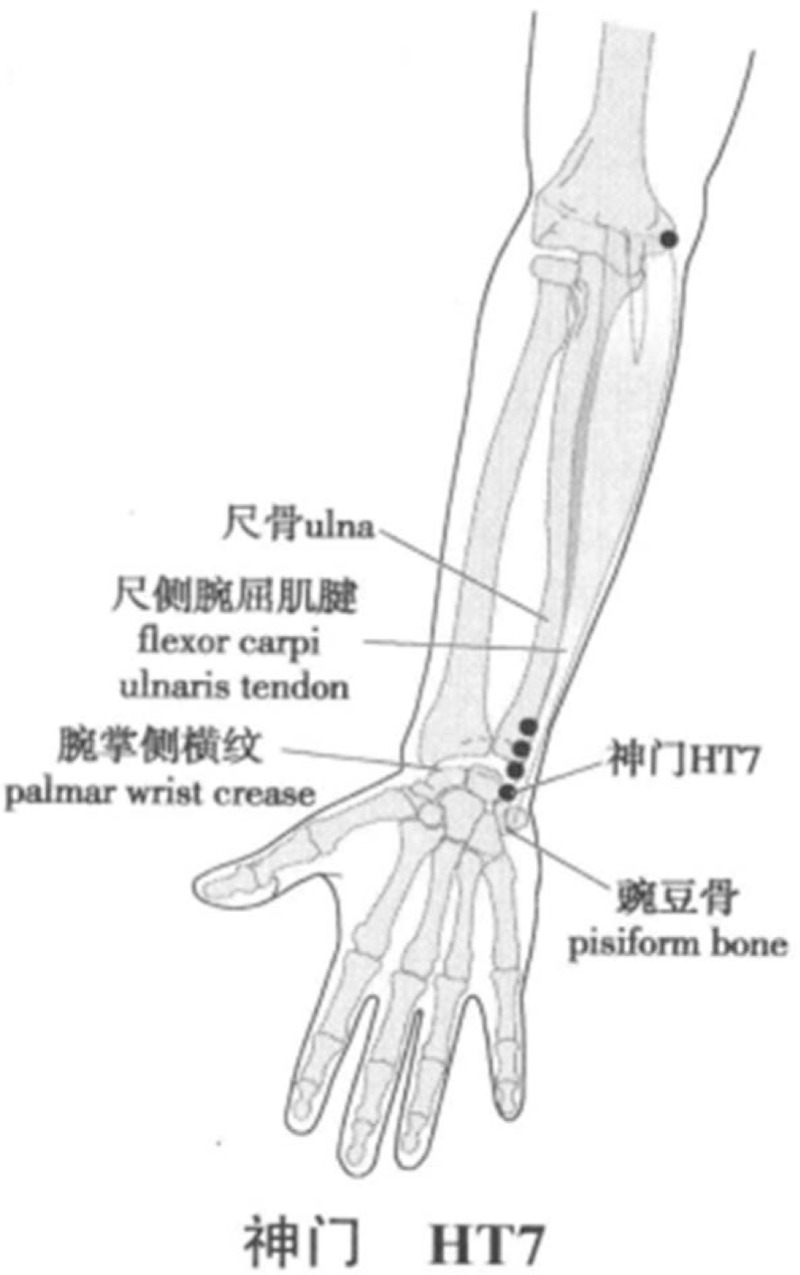
Location of Shenmen (HT 7) Acupoint. Longxiang H. WHO standard acupuncture point locations in the Western Pacific region (Chinese-English bilingual edition) [M]. Beijing: People's Medical Publishing House, 2010.^[16]^

The rTMS therapeutic instrument (CCY-II, Wuhan yiruide medical products New Technology Co., Ltd., Wuhan) was used. According to the theory of traditional Chinese medicine, magnetic stimulation of left HT7 point was selected. After the preparation, the stimulation coil will be placed on the left HT7 acupoint (anteromedial wrist, radial to ulnar flexor tendon, on the metacarpal wrist fold) (Fig. 1). The setting parameters are: CTBS mode, total pulse number 1800,^[14]^ stimulation intensity 15%.^[15]^ Course of treatment: twice a day, with an interval of more than 4 hours, 5 days / wk, a total of 30 treatments.

#### 2.7.2 Control group

The control group was only given basic treatment.

#### 2.7.3 Healthy control group

There was no intervention during the study period.

### 2.8 Adverse events observation

Local skin numbness around HT7 point may be related to rMS of HT7 point. During the study, a patient treatment log will be established to monitor all adverse reactions during treatment and follow-up, and appropriate intervention will be carried out.

Patients with severe insomnia during the study were given dexzopiclone (3 mg QN). However, subjects who could not tolerate 80% of the rMS intensity and / or took more than twice a week of dexzopiclone were considered withdrawn.

In case of serious adverse reactions during the treatment, report to the principal investigator immediately, record the details in the patient treatment log and take corresponding treatment measures. If the subject or researcher thinks that the patient cannot tolerate these adverse reactions, the trial can be terminated and the subject will withdraw from the study.

### 2.9 Primary outcomes

The cognitive function of the patients was evaluated on the day of enrollment and the 20th day after treatment:

1. 1-back working memory;
2. Rey osterieth complex figure test;
3. Hanoi tower test.

### 2.10 Secondary outcomes

The use of the following scales was evaluated on the day of enrollment and the 20th day after treatment:

(1) insulin severity index;
(2) Becker depression scale;
(3) Becker anxiety scale.

The patients’ mood, sleep, and diet were followed up through the treatment log. All adverse reactions and causes of shedding will be recorded in case report form (CRF) in time.

### 2.11 Estimation of sample size

The sample size was the minimum number of 30 cases in clinical trials. Considering the loss rate of 20%, 36 cases of N1 = N2 = 72 cases were selected in this study, and 36 cases of healthy controls were included.

### 2.12 Randomization

This study is a randomized controlled open trial. The method of randomization and allocation concealment is to generate 72 serial numbers according to the order of visit. The first group was randomly assigned to the first group, and the second group was randomly assigned to the last group. The randomized grouping scheme was kept in a sealed envelope, and the envelopes were opened in turn according to the order of admission, and the grouping of patients was determined according to the allocation scheme in the envelope.

### 2.13 Statistical analysis

According to the situation of data collection, data analysis will be based on the principle of intention to treat analysis and / or per protocol analysis. We will also evaluate the group effect by comparing the analysis results of the above 2 data sets to evaluate the accuracy of our analysis results.

All data will be analyzed by third-party statisticians using SPSS 23.0 (v23.0, SPSS Inc, Chicago, IL). *P* < .05 was significant. Descriptive statistics will be carried out by group and time for average, standard deviation, maximum value, minimum value, and so on.

The data in accordance with the protocol set were selected for analysis. If the data obey normal distribution, they can be described by means, and standard deviation. If the variance is uniform, the analysis of variance of complete random design can be used to compare the effect indexes. If the variance is not uniform, the rank sum test of multi group comparison of complete random design can be used. If the data does not obey normal distribution, it can be described by median and interquartile distance. The hypothesis test used the bilateral test uniformly, with *P* < .05 as the statistical significance.

#### 2.13.1 Primary outcomes

On the day of admission and the 20th day of treatment, the patients will be evaluated with

1-back working memory,

Rey osterieth complex figure test and

Hanoi tower test, and the obtained data will be used by rank sum test or Chi-squared test.

After statistical analysis between groups, the significant influence between groups will show that the 1-back working memory, Rey osterieth complex figure test, Hanoi Tower test there are differences.

#### 2.13.2 Secondary outcomes

The patients were evaluated with insignia severity index, Becker depression scale and Beck Anxiety Scale on the day of enrollment and the 20th day of treatment, and the data were analyzed by rank sum test or Chi-squared test.

### 2.14 Safety evaluation

Adverse events recorded in the patient's diary will be analyzed as a multiplicative variable. The number and percentage of AE patients will be calculated and compared using Chi-squared test

### 2.15 Quality control

During the whole treatment and follow-up process, patients’ withdrawal and the reasons for withdrawal will be recorded in time. In order to ensure the test quality, the quality supervisor will regularly check all process details and check the authenticity of the data. In addition, the scientific research department of Shaanxi Provincial Hospital of traditional Chinese medicine will be invited to manage the data independently.

As differences between doctors may lead to errors, each evaluation of the scale will be performed by a designated physician trained in the same evaluation criteria. The 2 doctors in charge of the treatment will receive special training on research and operation, including the use of RMS and acupoint positioning. In order to record the patient's treatment and compliance, we made a record card for the patient, including the treatment date, personal information and signature after each treatment.

## 3 Discussion

Sleep is an important part of human life activities. It can eliminate fatigue, enhance immune function, regulate emotion, and regulate cognitive function. Ci can not only lead to sub-health status, but also lead to damage in many cognitive fields, which brings serious harm to patients and society. No matter whether CI has cognitive impairment or not, cognitive function has been impaired to a certain extent.^[2]^

Neurocognitive deficits after CI are caused by neurobiological changes.^[17]^ Compared with healthy people, the regional homogeneity of the upper right medial prefrontal cortex was significantly different in CI patients; and the more serious the cognitive impairment, the greater the homogeneity difference of the dorsolateral cortex of the right prefrontal cortex.^[18]^ A voxel based fMRI morphometric study found that the volume of the left orbitofrontal Gray matter in insomnia patients decreased, and it was closely related to the subjective severity of insomnia.^[19]^ The microstructure of prefrontal cortex in insomnia patients is damaged, which affects the emission of brain nerve impulses and signal transmission, resulting in insufficient activation of cognitive processing and dysfunction of brain functional connectivity, which may be an important neurobiological process of Ci and its accompanying cognitive impairment.

RTMS and traditional Chinese medicine acupuncture and moxibustion are commonly used in CI treatment, and have significant curative effect. Compared with rTMS, RMS has fewer contraindications. Compared with acupuncture, RMS has the advantages of noninvasive and painless, which can reduce the pain of patients and increase the compliance of patients. In this study, rMS was combined with acupuncture and moxibustion of traditional Chinese medicine, and HT7 was used to intervene cognitive impairment of CI. The purpose of this study is to observe the clinical efficacy of magnetic stimulation of HT7 on cognitive impairment in patients with CI through the evaluation of cognitive function, sleep quality, and emotion of CI patients, and analyze the possible mechanism.

Our scheme is only a pilot study, so there may be problems in sample size and observation time. In the course of the experiment, we will try our best to reduce the deviation that affects the research results. Finally, we intend to confirm that magnetic stimulation of HT7 can be used as an alternative therapy to improve cognitive impairment of CI.

## Author contributions

Jie Yuan and Yaling Lei designed this study together. Penglong Yu, Jie Yuan, and Yimeng Chen drafted the protocol. Jie Chen performed the statistical analysis. Yaling Lei was responsible for the writing revision. All authors read and approved the final manuscript.

**Acupuncture treatment:** Yuan Wang

**Data collection:** Yuan Wang, Fan Luo, Pei Wang

**Formal analysis:** Penglong Yu, Yuan Zhao, Jie Chen.

**Funding acquisition:** Ya ling Lei

**Methodology:** Jie Yuan, Jie Chen

**Project administration:** Yongxiang Gao, Jie Yuan

**Project coordination:** Yongxiang Gao, Yimeng Chen

**Resources:** Yuan Wang.

**Supervision:** Yongxiang Gao.

**TMS treatment:** Yuan Zhao

**Writing – original draft:** Jie Yuan, Yimeng Chen, Penglong Yu.

**Writing – review & editing:** Jie Chen, Yaling Lei.
